# Dynamic Stromal Alterations Influence Tumor-Stroma Crosstalk to Promote Pancreatic Cancer and Treatment Resistance

**DOI:** 10.3390/cancers13143481

**Published:** 2021-07-12

**Authors:** Kendelle J. Murphy, Cecilia R. Chambers, David Herrmann, Paul Timpson, Brooke A. Pereira

**Affiliations:** 1 The Kinghorn Cancer Centre, Garvan Institute of Medical Research, Sydney, NSW 2010, Australia; k.murphy@garvan.org.au (K.J.M.); c.chambers@garvan.org.au (C.R.C.); d.herrmann@garvan.org.au (D.H.); 2 St. Vincent’s Clinical School, Faculty of Medicine, University of New South Wales, Sydney, NSW 2010, Australia

**Keywords:** pancreatic cancer, stroma, tumor microenvironment, extracellular matrix, cancer-associated fibroblasts, biomechanics, stromal targeting

## Abstract

**Simple Summary:**

Pancreatic ductal adenocarcinoma (PDAC) is one of the most lethal solid malignancies, with a five-year survival rate of only ~10%. Pancreatic tissue becomes increasingly fibrotic (known as desmoplasia) during cancer development and progression. This extensive, heterogeneous reaction is largely mediated through the actions of stromal cells such as cancer-associated fibroblasts (CAFs). In this review, we will discuss how heterotypical reciprocal tumor-stromal and tumor-immune cell interactions in the pancreatic tumor microenvironment (TME) can both promote and restrain PDAC development and progression, with particular focus on the role of extracellular matrix (ECM) in potentiating tumor cell proliferation, survival, metastasis, and treatment resistance. We also give a snapshot of the current and emerging stromal co-therapies used in combination with chemotherapy or immunotherapy to treat this highly deadly disease.

**Abstract:**

Many cancer studies now recognize that disease initiation, progression, and response to treatment are strongly influenced by the microenvironmental niche. Widespread desmoplasia, or fibrosis, is fundamental to pancreatic cancer development, growth, metastasis, and treatment resistance. This fibrotic landscape is largely regulated by cancer-associated fibroblasts (CAFs), which deposit and remodel extracellular matrix (ECM) in the tumor microenvironment (TME). This review will explore the prognostic and functional value of the stromal compartment in predicting outcomes and clinical prognosis in pancreatic ductal adenocarcinoma (PDAC). We will also discuss the major dynamic stromal alterations that occur in the pancreatic TME during tumor development and progression, and how the stromal ECM can influence cancer cell phenotype, metabolism, and immune response from a biochemical and biomechanical viewpoint. Lastly, we will provide an outlook on the latest clinical advances in the field of anti-fibrotic co-targeting in combination with chemotherapy or immunotherapy in PDAC, providing insight into the current challenges in treating this highly aggressive, fibrotic malignancy.

## 1. Introduction

Pancreatic ductal adenocarcinoma (PDAC) is the most common type of pancreatic cancer representing 95% of all patients and remains one of the most lethal forms of human cancer worldwide, with >90% of patient deaths occurring within one year of diagnosis [1]. PDAC is projected to increase to the second-deadliest cancer type in the US by 2030, unless treatment options are improved [1,2]. One of the reasons PDAC has such a poor prognosis is because ~80–90% of patients first present to the clinic with advanced-stage, invasive or metastatic disease, which in most cases does not qualify for surgical removal of the tumor [1]. As a result, surgical resection with curative intent is only available to around 10–20% of patients [1,3], and of those who undergo curative surgery, 80% will eventually relapse and succumb to the disease [4]. As surgical resection is only available to a small proportion of patients, the majority of PDAC patients will be subjected to other therapies including radiation therapy, chemotherapy, chemoradiation therapy and more recently targeted therapies. For more than 20 years the standard-of-care for advanced PDAC has been gemcitabine [5], a nucleoside analogue that inhibits DNA synthesis inducing a caspase-driven apoptotic cascade, leading to cancer cell death [6]. Recently the addition of Abraxane (Nab-paclitaxel) to gemcitabine and the drug combination FOLFIRINOX have improved median survival in PDAC from six months (gemcitabine monotherapy) to 8.5 and 11.1 months, respectively [7,8,9]. Abraxane inhibits the depolymerisation of microtubules to arrest mitosis and induce cancer cell death [10], while FOLFIRINOX is a cocktail of cytotoxic drugs, including fluorouracil, irinotecan and oxaliplatin as well as leucovorin, which has been proven to be effective in PDAC [7,11]. Although FOLFIRINOX shows modest improvements in patient survival when compared with gemcitabine/Abraxane combination therapy, it also exhibits increased associated toxicities therefore mostly limiting its use to ‘fit’ patients [12]. Thus, although significant improvements in PDAC treatment have been made, the overall five-year survival rate has remained largely unchanged for 40 years and novel therapeutics are desperately required.

PDAC is thought to arise in the ductal cells of the exocrine glands and is widely accepted as progressing through a number of pre-invasive pancreatic intraepithelial neoplasm (PanIN) stages before the development of PDAC. Several large-scale epidemiological and genetic studies have recently led to the identification of a large range of potential oncogenic drivers of the disease [13,14,15]. Despite this high genetic diversity, mutation and activation of the KRAS oncogene is almost always required for the initiation of PanINs, with KRAS mutations found in 90% of all PDAC samples, driving cancer cell proliferation and survival [16,17,18]. Whilst activating mutations in KRAS are responsible for initiation in the majority of PDAC cases, in vitro and in vivo studies in both human and mouse models have identified that disease progression requires subsequent mutations and/or loss of gene function such as in Trp53 (p53), SMAD4, CDKN2A and BRCA2, triggering further aberrant cell survival and proliferation, and overcoming KRAS-induced senescence [18,19,20,21,22,23,24,25,26]. The vast heterogeneity of PDAC tumors has led to several key sequencing and proteomic studies aimed at matching individual molecular PDAC profiles with therapeutically targetable subtypes [13,14,15], as previously established for other cancer types such as breast cancer. 

**Figure 1:**
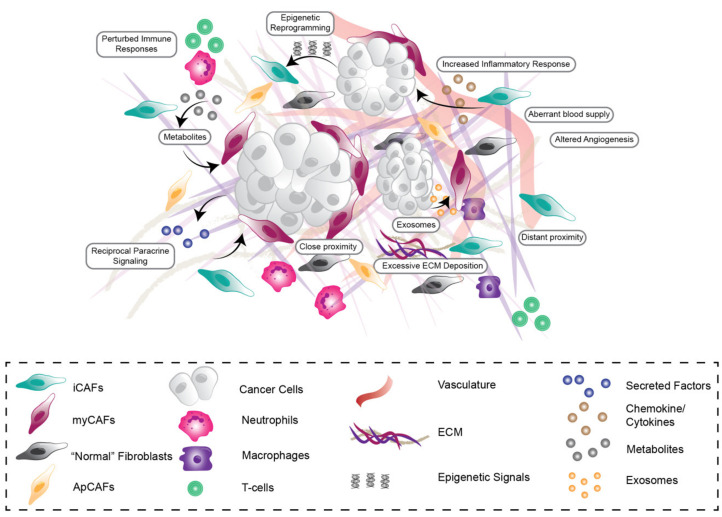
Stromal Heterogeneity in the pancreatic tumor microenvironment. The pancreatic tumor microenvironment is highly heterogenous, consisting of cancer cells, activated cancer-associated fibroblast (CAF) subpopulations, increased deposition, remodeling and degradation of extracellular matrix (ECM), aberrant vasculature and impaired immune cell response. CAF subpopulations are influenced by direct-, short- and long-range growth factor (e.g., FGF1), metabolic, chemokine (e.g., CXCL12) and exosome paracrine signalling (indicated by arrows between different cell types and subpopulations) as well as epigenetic regulation via cancer cells, immune cells, vasculature and neighbouring CAFs.

In addition to this high genetic and molecular diversity, PDAC is also one of the most stromally-dense cancer types across all malignancies, with stromal desmoplasia or fibrosis accounting for up to 90% of the total tumor volume [27]. This desmoplasia is characterized by short and long-range reciprocal interactions between cancer cells and stromal components, including cancer-associated fibroblasts (CAFs), endothelial and immune cells as well as extracellular matrix (ECM), which influence all stages of tumorigenesis as well as therapeutic efficacy and resistance (Figure 1) [28,29]. PDAC desmoplasia involves the recruitment and activation of CAFs, excessive ECM deposition, remodeling and degradation [30,31], increased inflammatory responses [32], aberrant immune responses as well as altered angiogenesis and blood supply [33,34], which can ultimately contribute to compromised drug delivery and efficacy (Figure 1). CAFs are one of the most abundant and active components of the PDAC microenvironment and are the main source of ECM components, such as the various types of collagens, proteoglycans, glycoproteins and hyaluronic acid (HA) [30], which have all previously been associated with PDAC tumorigenesis. Overall, CAF-derived ECM is a dominant force in early tumor progression as well as later invasion, metastasis, and treatment resistance [31]. The prominent role of CAFs in the microenvironment has long been assumed as an ‘Achilles’ heel’ in PDAC progression and has therefore led to numerous attempts to target them in combination with other treatment options. Surprisingly, previous work in mouse models has shown that complete depletion of stromal CAFs can lead to poorly differentiated and aggressive tumors resulting in shorter survival, indicating the stroma can also restrain cancer progression in addition to its well established pro-tumorigenic roles [35,36]. Additionally, it was recently reported that myofibroblast-specific deletion of Collagen I (Col I), one of the most abundant ECM proteins in PDAC, results in the acceleration of PanIN progression and PDAC emergence. Furthermore, this loss of Col I promoted an immunosuppressive TME, thereby decreasing anti-cancer immunity and overall survival [37]. Moreover, several PDAC clinical trials reflected these unexpected pre-clinical findings where complete inhibition of stromal fibrosis via targeting of the hedgehog pathway resulted in either no added survival benefit for patients or potentially harmful side effects over gemcitabine or FOLFIRINOX alone [38,39,40]. These findings suggest that a more fine-tuned, nuanced approach is required to effectively target the stroma in PDAC without causing negative side effects. 

CAFs are thought to originate from a diverse range of stromal cell progenitors including pancreatic stellate cells (PSCs) [41,42,43,44,45], mesothelium [46], resident fibroblasts [47], mesenchymal stem cells (MSCs) [48] and bone marrow-derived stem cells [49]. Common “pan-CAF” markers include COL1A1/2 (collagen I alpha 1/2), DCN (decorin), PDPN (podoplanin), FAP (fibroblast activation protein) and VIM (vimentin) [32,50]. Recently however, the traditional view of a uniform CAF cell type within a tumor has been re-evaluated. Rather, CAFs are a highly dynamic and heterogenous cell population that can be both tumor-promoting and tumor-restraining [28]. Moreover, it is now well appreciated that CAFs can rapidly respond to disruptions in tissue homeostasis, signalling and mechanical changes in their environment [28,29]. Recently with the advent of single cell RNA sequencing (scRNASeq), distinct subtypes of CAFs have been identified, in both mouse models of PDAC and human PDAC tissue [32,50,51,52], with new subpopulations emerging continually. In the KPC mouse model (KRAS^G12D^; p53^R172H^; PdxCre) and human PDAC specimens, distinct fibroblast subpopulations have been identified including myofibroblasts (myCAFs), inflammatory fibroblasts (iCAFs) and antigen presenting CAFs (apCAFs) [32,50] (Figure 1). In addition to pan-CAF markers, myCAFs exhibit high expression of alpha-smooth muscle actin (α-SMA) and low levels of interleukin-6 (IL-6) as well as low levels of other inflammatory interleukins such as IL-11 and LIF (leukaemia inhibitory factor), whereas iCAFs exhibit lower levels of α-SMA and high levels of IL-6, IL-11 and LIF [32,50]. Meanwhile, apCAFs express pan-CAF markers as well as a range of genes relating to the MHC class II family including H2-Aa and H2-Ab1, as well as distinct pro-tumorigenic and pro-inflammatory genes such as SAA3 and SLPI [50]. The progenitors of these CAF subpopulations are yet to be fully elucidated, however Garcia et al. (2020) recently reported that some myCAFs could be derived from GLI1+ fibroblast progenitors [47]. CAF subpopulations also exhibit some spatial distinctions, where myCAFs tend to sequester adjacent to tumor cells and iCAFs tend to be located distally from tumor cells (Figure 1). Moreover, it has been shown ex vivo that apCAFs could be converted to myCAFs by altering the CAF culture conditions, demonstrating that CAFs can dynamically switch subtypes according to environmental cues [50]. This, together with the spatial organization of myCAFs and iCAFs, although not indisputable, suggests that CAFs may exhibit different phenotypes dependent on their environment, including their proximity to cancer cell stimuli. Furthermore, recent work by Feldmann et al., (2021) has shown that the transcription factor PRXX1, is in part responsible for tuning CAF activation and plasticity in PDAC tumors [53]. In this study, deletion of PRXX1 drove the expansion of tumor-restraining CAFs, leading to increased tumor differentiation, as well as improved sensitivity to gemcitabine chemotherapy and reduced tumor dissemination [53]. Overall, understanding more about the transcriptional and microenvironmental drivers of CAF phenotype and function in PDAC will be critical to understanding their context-dependent influence on all stages of pancreatic tumorigenesis and will inform how best to target these pro-tumorigenic features.

## 2. Stromal Features Can Influence and Predict Outcomes in PDAC

As previously mentioned, PDAC is a highly heterogeneous disease, where a wide range of distinct genetic as well as epigenetic and microenvironmental alterations govern the progression and stratification of the disease. Therefore, categorizing PDAC tumors into therapeutically actionable subtypes may be a valuable strategy. Previous genomic studies have suggested that PDAC tumors could be assigned to predominantly “classical”, “squamous”, “progenitor” or “basal-like” subtypes [13,14,15]. Although classification of these subtypes has begun to pave the way for personalised medicine approaches in PDAC, they are largely focused on the epithelial compartment of the disease, with only limited focus on the stromal contributions. Indeed, it is becoming increasingly apparent that inclusion of the stroma in genomic analyses can enhance the accuracy in predicting clinical prognosis. For example, Moffitt and colleagues (2015) virtually dissected tumor, stromal and “normal” gene expression data from a large cohort of pancreatic specimens including 145 primary tumor sites, 61 metastatic sites, 17 cell lines, 46 wildtype pancreata and 88 distant site-adjacent “normal samples”. In this study, the authors identified a ‘normal’ and ‘activated’ stromal signature, with the latter being poorly prognostic [54]. In addition, this activated stromal subtype was characterised by expressing a diverse set of genes, including SPARC, periostin, Wnt family members and MMP9 (gelatinase B) as well as macrophage-associated genes, suggesting that this subtype might exhibit a pro-inflammatory stromal response that impeded patient survival [54]. Although this study included a large number of diverse specimens, it focused on the virtual dissection of bulk tumor samples and therefore may have suffered from a biased selection of tissue compartments in silico. Maurer et al., (2019) expanded upon this previous work by using laser capture microdissection to physically isolate pathologically verified epithelial and stromal sections from over 60 different PDAC patients [55]. These specimens then underwent RNAseq, with the data obtained used to examine three independent PDAC cohorts (UNC, TCGA and ICGC) via a machine learning algorithm [55]. Converse to Moffit et al., (2015), this study modified the classification of the two previously described stromal PDAC subtypes (‘normal’ versus ‘activated’ stroma) to an ‘immune-rich’ group, which showed increased expression of immune-related signalling proteins, and an ‘ECM-rich’ group, which was enriched for gene sets associated with ECM deposition, remodeling and interaction [55]. From these new classifications, the authors found that the ECM-rich group was associated with decreased survival compared to the immune-rich group [55]. It is clear from these large-scale studies that analysis of both the epithelial and stromal contributions in PDAC can help to predict clinical outcomes more accurately for individual patients.

The stroma itself has also been shown to influence the molecular (epithelial) subtype of PDAC tumors. In a study by Miyabayashi et al., (2020), patient-derived organoids were specifically injected into either the pancreatic ducts or pancreatic interstitium of host mice [56]. Traditionally, most orthotopic models of PDAC do not inject into the ducts but are typically non-specifically injected into the interstitial tissue. Strikingly, downstream sequencing analysis showed that the tumors injected specifically into the ducts displayed the progressive switching of two subtypes, where the tumors displayed either “classical/progenitor” features with low stromal activation, or squamous/basal-like characteristics with high stromal activation [56]. Meanwhile, tumors injected into the interstitial tissue were uniformly all squamous/basal-like with high stromal activation [56]. This study exemplifies the need to alter or improve current PDAC models to better represent stromal microenvironments, thereby increasing the physiological relevance of new pre-clinical findings. A detailed review of stromal PDAC biology models can be found summarized in [57]. The reciprocal relationship between molecular subtype and stroma was further highlighted by Somerville et al., (2020), who showed that PDAC cells which specifically display a squamous phenotype can trigger the secretion of inflammatory mediators, promoting stromal inflammation and CAF activation [58]. Together this highlights the influence of the reciprocal feedback and dynamic signalling between tumor and stroma on the previously described subtypes of PDAC.

Importantly, the stromal impact on PDAC progression and response to treatment is not only limited to the primary tumor site but can also be assessed in the periphery. Here, stromal or ECM-based biomarkers are emerging as a novel approach to identify disease progression or treatment response in patients. PDAC has a paucity of specific and sensitive biomarkers, with carbohydrate antigen 19-9 (CA19-9) being the only routine biomarker used in the clinic [59,60]. Unfortunately, CA19-9 is not highly specific to PDAC (only found in the serum of ~75% of patients) and is also secreted in other conditions such as benign pancreatic diseases (e.g., pancreatitis) and other cancers [61]. Considering the stromal density and extensive ECM turnover in PDAC tumors, it is logical that using ECM-based biomarkers could potentially help during diagnosis of the disease. For example, Willumsen and colleagues showed in a Phase III clinical trial of PDAC that pre-treatment serum levels of proteolytically degraded Col I, Col III, Col IV fragments and PRO-C3 (a pro-peptide of Col III) were significantly upregulated in the serums of PDAC patients [62]. These markers were used as a surrogate readout of PDAC desmoplasia and ECM remodeling, and all showed that low expression was associated with increased survival [62]. 

Furthermore, extracellular vesicle (EVs), which carry nucleic acids, lipids and proteins, have gained attention in cancer diagnostics and prognostics due to their established ‘messenger’ role from the primary tumor to other parts of the body, including putative secondary sites [63,64,65,66,67,68]. For example, in a large-scale screen of EV proteins from PDAC and lung adenocarcinomas, several PDAC-specific EVs could be identified in both tissue biopsies and plasma from patients to a high level of sensitivity and specificity [68]. EV biomarkers of note include the stromal proteins versican (VCAN) and thrombospondin 2 (TSP2), which were part of a highly accurate (~90% sensitivity/95% specificity) 16 EV pan-cancer signature [68]. More recently, Huang and colleagues (2020) found four secreted EV proteins that were enhanced in patient-derived xenograft (PDX) organoids, and then validated these proteins in plasma samples from PDAC patients [69]. Strikingly, they found two EV proteins, ANXA11 and GPC4, that were significantly upregulated in PDAC compared to patients presenting with chronic pancreatitis (a risk factor for subsequent PDAC diagnosis) [69], highlighting the need for further research into the PDAC secretome to better understand patient prognosis as well as to improve treatment opportunities. Overall, development of both stromal (and non-stromal) peripheral biomarkers in PDAC is of utmost importance, considering clinical presentation of the disease is often late, when metastasis has already occurred. Further research in this area could help improve patient outcomes and response to subsequent therapies.

It is becoming increasingly evident that the stromal contribution to disease progression can also be influenced by immune cells. For example, Mahajan et al., (2018) combined a range of immune-related markers (CD3, CD4, CD8, CD68 and CD206) with stromal markers to establish a histological signature from a tissue microarray (TMA) cohort of 93 patients which was prognostic of progression-free survival (PFS). In fact, the authors found that a PDAC stroma with high α-SMA+ cells and low collagen content favoured an immunosuppressive TME, with increased numbers of pro-tumorigenic M2 macrophages and decreased cytotoxic CD8+ T cells [70]. Conversely, high α-SMA and high collagen correlated with increased PFS, exhibiting higher numbers of cytotoxic CD8+ T cells and anti-tumorigenic M1 macrophages [70]. Interestingly, in this study the stromal composition alone (without an association with inflammatory cell markers), did not correlate significantly with PFS [70]. This study indicates that immune cell composition may present an additional layer that influences whether the stroma has a pro- or anti-tumorigenic effect in PDAC. In contrast, there are several studies that do show that direct effects of the stroma on patient outcomes including a recent study by Tahkola et al. (2021), which reported that stromal HA accumulation is poorly prognostic for PDAC patients [71,72]. Moreover, stromal features may also influence treatment approaches in PDAC patients. For example, Ogawa et al., (2021) used multiplexed fluorescence immunohistochemistry (IHC) to identify three distinct stromal PDAC subtypes known as “collagen-rich”, “FAP-rich” and “α-SMA-rich” [73].

Moreover, stromal features may also influence treatment approaches in PDAC patients. For example, Ogawa et al., (2021) used multiplexed fluorescence immunohistochemistry (IHC) to identify three distinct stromal PDAC subtypes known as “collagen-rich”, “FAP-rich” and “α-SMA-rich” [73]. Interestingly, the proportion of FAP+ CAFs was inversely correlated with the ratio of intra-tumoral CD8+ T cells (relative to the border), suggesting that FAP-dominant fibroblasts may in part be responsible for the spatial exclusion of cytotoxic CD8+ T cells [73]. This further indicates that FAP-rich stroma might be more resistant to immunotherapy such as checkpoint blockade, as high numbers of infiltrating CD8+ immune cells in the local TME are generally required for immunotherapy to be effective. Overall, these studies highlight how stromal features (including infiltrating immune cell populations) can reciprocally influence clinical outcomes and treatment strategies and that both the stromal and epithelial contributions could be the key to better stratifying PDAC patients for treatment.

## 3. Heterotypic Reciprocal Tumor-Stroma Signalling Drives PDAC Development, Progression, and Therapy Resistance

**Figure 2:**
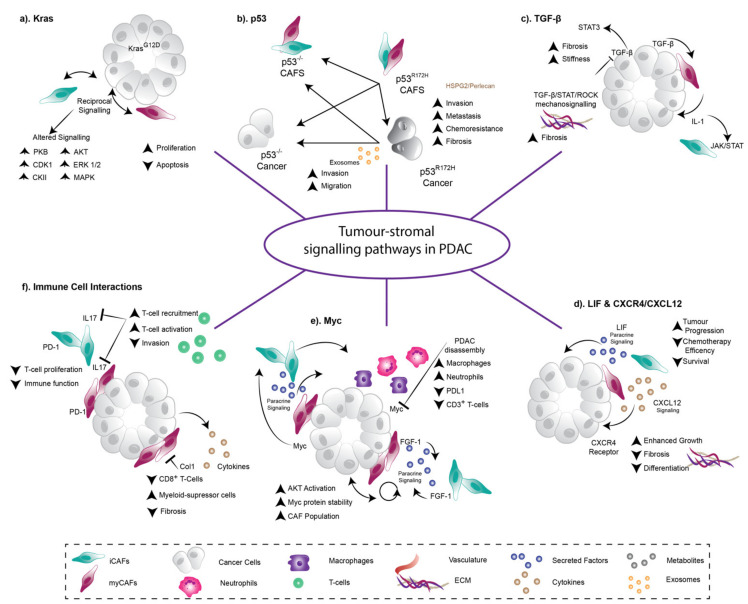
Heterotypic tumor-stroma signalling drives pancreatic cancer development, progression, invasion, and metastasis. (**a**) Reciprocal Kras^G12D^ signalling between tumor and stromal cells regulates signalling axes, which influence cancer cell proliferation and apoptosis [74]. (**b**) Cancer cells with distinct p53 mutations can modulate neighbouring and distant fibroblasts to establish a fibrotic, pro-invasive and pro-metastatic environment [29, 75]. (**c**) TGF-β signalling via STAT3 increases desmoplasia and stiffening of the tumor ECM and is fundamental in the distinct formation of myCAFs and iCAFs. Additionally, tumor secreted TGF-β acts on adjacent myCAFs and antagonises IL-1 secretion to further activate JAK/STAT signalling [76]. (**d**) Chemokine receptors CXCR4 play a crucial role in stromal desmoplasia. Here, signalling enhances tumor growth whilst also decreasing fibrosis and cell differentiation [77]. Similarly, LIF paracrine signalling promotes tumor progression whilst also decreasing chemotherapeutic efficiency [78]. (**e**) The transcription factor, Myc mediates the expression of multiple genes to coordinate cell proliferation. In the stromal compartment, early PanIN activation triggers paracrine signalling, which then induces stromal and immunological changes, driving disease progression [79]. (**f**) Within the pancreatic tumor microenvironment, innate and adaptive immune cells affect disease progression. Knockdown of Interleukin-17 in fibroblasts leads to an anti-tumor immune microenvironment, including increased cytotoxic T-cell recruitment [80]. Conversely, high PD-1 expressing fibroblasts contributed to a diminished immune function and T-cell proliferation [81].

In many solid tumors including PDAC, tumor cells and stromal cells participate in dynamic, context-dependent paracrine signalling, which can both promote and impede cancer development, progression, and response to treatment. In this section we will explore some key signalling pathways which govern stromal phenotype and function in PDAC including KRAS, p53, TGF-β, Myc and interleukins (Figure 2).

KRAS mutations are often considered the major driver during PDAC initiation being present in approximately 90% of PDAC patient tumors [13]. In 2016, Tape and colleagues reported that PDAC tumor cell mutant KRAS (KRAS^G12D^) signals reciprocally through stromal cells, to subsequently enhance tumor cell function [74]. That is, when KRAS is mutated in pancreatic cancer cells, they signal autonomously to increase cell proliferation via activation of extracellular signal-regulated protein kinase 1 and 2 (ERK1/2) and increased phosphorylation of several kinases such as mitogen-activated protein kinase (MAPK), cyclin-dependent kinase 1 (CDK1) and casein 2 kinase (CKII) [74] (Figure 2a). In addition to this cell autonomous signalling, KRAS mutant cancer cells can also signal to adjacent fibroblasts to change their phenotype and function [74]. From here, these hijacked fibroblasts signal back to the cancer cells, triggering signalling cascades in the cancer cells such as protein kinase B (PKB; also known as AKT) signalling, which would otherwise not be activated [74] (Figure 2a). The result of these heterocellular reciprocal interactions is that oncogenic KRAS signalling bypasses “tumor cell only” signalling, potentiating further tumorigenesis [74] (Figure 2a). Furthermore, Ischenko et al., (2021) recently reported that mutant KRAS is critical to immune evasion in PDAC tumors. In this study, mutant KRAS was inactivated in KRAS^G12D^, p53KO pancreatic tumor cells to show that upon loss of the KRAS mutation, cells still retained tumorigenic capacity, but lost their ability to evade the adaptive immune system [82]. Therefore, KRAS mutation can have significant pro-tumorigenic effects in the TME beyond PDAC initiation via both stromal cells and the immune system. 

Similarly, alterations in the tumor suppressor gene p53 can have extensive effects on the pancreatic TME to potentiate invasion and metastasis. In 2019, we reported that KRAS mutant cancer cells with a gain-of-function p53 mutation can “educate” adjacent CAFs via short and long range NFκB/TNFα signalling, driving the establishment of a pro-metastatic and chemoresistant environment by secreting perlecan (a basement membrane protein) [29] (Figure 2b). Likewise, Novo et al., (2018) reported that pancreatic cancer cells with a mutant p53 phenotype can activate fibroblasts to be pro-invasive via exosomal secretion of a sialylated glycoprotein called podocalyxin (PODXL), while p53 null-derived exosomes could not [75] (Figure 2b). Strikingly, it was also shown that exosome-derived PODXL affects ECM organisation and remodeling in the lungs of mice enhancing metastatic colonization [75]. These studies demonstrate the influence of p53 mutational status on disease progression and therapeutic response further highlighting the heterogeneous nature and influence of different stromal populations on tumor behavior [28,29].

Members of the transforming growth factor-β (TGF-β) family of secreted proteins bind to TGF-β receptors on the cell surface to regulate gene expression via SMAD phosphorylation. TGF-β signalling has been shown to exhibit both tumor-suppressing and tumor-supporting roles in PDAC, depending on the tumor stage, the differentiation status of the tumor, and cell type [83]. For example, Laklai et al., (2016) reported that human PDAC tumors with loss of epithelial TGF-β signalling develop a STAT3-mediated desmoplastic and mechanically stiff stroma, activated through increases in epithelial actomyosin tension and elevated β1-integrin mechanosignalling (Figure 2c) [84]. Furthermore, ablating STAT3 in this context resulted in normalization of tissue stiffness and tension, slowing tumor progression in PDAC mouse models (Figure 2c) [84]. A later study by Pinho et al., (2018) showed that, in the context of pancreatic tissue injury, the neuronal axon-pathfinding ROBO-SLIT pathway can regulate TGF-β signalling, leading to distinct stromal remodeling in advanced PDAC mouse models [85]. After injury, loss of ROBO2 in pancreatic epithelial cells caused enhanced myofibroblast activation and collagen crosslinking, as well as a pronounced pro-tumorigenic immune response [85]. Strikingly, abrogating TGF-β signalling via the TGF-β receptor I small molecule inhibitor galunisertib normalized these effects in mouse models [85]. Furthermore, Ligorio et al., (2019) reported that CAF-derived TGF-β can drive the expansion of a highly proliferative PDAC tumor cell subpopulation that can readily undergo epithelial-to-mesenchymal transition (EMT) arguing for a pro-tumorigenic role of TGF-β signalling in PDAC [86]. Later work showed that TGF-β is also fundamental for the formation of myCAFs and iCAFs in both mouse and human PDAC, through its activation of the IL-1/JAK/STAT signalling axis [76]. In a complex feedback loop, tumor-secreted TGF-β acts upon adjacent myCAFs, which in turn antagonises tumor secretion of IL-1 and subsequent activation of the JAK/STAT pathway in spatially distant iCAFs [76] (Figure 2c). It is clear that dysregulation of TGF-β signalling in PDAC is nuanced and highly context-dependent, with a duality of both anti- and pro-tumorigenic functions in vivo, highlighting the need for further studies prior to clinical intervention of this pathway in PDAC.

The CXCL12/CXCR4 signalling axis has also been shown to play a crucial role in PDAC stromal desmoplasia (Figure 2d) [77]. CXCR4 is the chemokine receptor for CAF-derived CXCL12 (also known as stromal cell-derived factor-1 (SDF1)) and is overexpressed by PDAC cells. In 2013, Feig et al., showed that targeting stromal CXCL12 improved T cell infiltration, thereby potentiating checkpoint inhibition in PDAC tumors [87]. More recently, Morita et al., (2020) showed that pancreas-specific CXCR4 deletion in the KPC mouse model resulted in reduced tumor cell-fibroblast crosstalk via CXCL12 (Figure 2d) [77]. This led to a significant reduction in pre-cursor PanIN lesions, but unexpectedly larger primary tumors [77]. Interestingly, these undifferentiated CXCR4-null tumors had higher tumor cellularity, with less ECM deposition and fewer stromal cells present, indicating that CXCR4 can be critical to the desmoplastic response in PDAC (Figure 2d). Further analysis revealed that CXCR4-null KPC tumor cells were more invasive and exhibited higher proliferative and migratory phenotypes than wildtype KPC cells (Figure 2d) [77]. It is possible that the chronic and permanent loss of tumor cell-derived CXCR4 in vivo reduced the number of tumor-restraining CAFs, while increasing the proportion of tumor-promoting CAFs. We propose a more nuanced, transient targeting of tumor-stromal interactions is required to potentially overcome this [88]. CXCR2 signalling via the myeloid cell population can also promote pancreatic tumorigenesis [89]. In a study by Steele et al., (2016), genetic or therapeutic loss of CXCR2 signalling resulted in reduced metastasis and prolonged survival, while also enhancing the efficacy of anti-PD-1 immunotherapy via increased cytotoxic T cell infiltration in mouse models [89]. Other secreted factors from activated stroma, such as LIF can also influence pancreatic tumorigenesis [78]. LIF is secreted by stromal cells in the pancreatic TME, which acts specifically on cancer cells to promote tumorigenesis (Figure 2d). It was previously shown that in mouse models of both genetic and pharmacological LIF blockade, loss of LIF resulted in significantly impaired tumor progression and augmented chemotherapy efficiency, prolonging survival by modulating cancer cell differentiation and EMT [78]. 

The pleiotropic transcription factor Myc mediates the expression of multiple genes, which coordinate several aspects of cell proliferation and is tightly controlled by mitogen availability in normal cells. However, upon activation of upstream oncoproteins in disease, aberrant Myc activation can drive cell proliferation and tumor growth. In a stromal context, Myc acts as a switch where its reversible activation in PanINs triggers the release of paracrine signals that coordinate stromal and immunological changes driving disease progression (Figure 2e) [79]. Promisingly, its deactivation in Myc-driven PDAC leads to disease regression, and reversal of Myc-driven tumorigenesis [79]. Furthermore, Myc is partially regulated by TME signalling where CAF-derived FGF1 can act as a paracrine regulator creating a permissive environment for AKT activation, which can stabilize Myc (Figure 2e) [90]. Here, patient specimens showed a strong correlation between Myc protein level and stromal CAF content, reasoning that oncogenic Myc levels may be a result of enhanced signalling from the TME (Figure 2e) [90].

The pancreatic stroma also harbors numerous innate and adaptive immune cells that potentially suppress anti-tumoral immune responses. In PDAC, the interleukin-17 (IL17) family is involved in multiple aspects of disease progression including neoplastic cell transformation (Figure 2f) [80]. Interestingly, genetic knockdown of IL17A in fibroblasts decreases their pro-tumorigenic functions and results in a conversion of the traditionally immunosuppressive TME into an anti-tumoral one (Figure 2f) [80]. Specifically, Mucciolo and colleagues observed changes in cytokines and chemokines produced by IL17A negative CAFs which lead to increased cytotoxic T cell recruitment and restrained tumor invasion (Figure 2f) [80]. Additionally, CAFs have been shown to have a role in shaping the immune system, whereby fibroblasts isolated from PDAC tumors of patients undergoing surgical resection, expressed higher levels of immunosuppressive PD-1 ligands compared to normal skin fibroblast of healthy individuals (Figure 2f) [81]. Here, CAFs are shown to inhibit T cell proliferation as well as induce immune checkpoint expression on T cells, which could contribute to a diminished anti-cancer immunity (Figure 2f) [81]. Such studies provide insight into the role of immune cell networks in PDAC progression and highlight the potential to improve immunotherapies in this disease.

## 4. Biomechanics Can Regulate PDAC Cell Fate

The biochemical and protein compositions of normal tissues have been well documented and are extensively recognized as regulators of cell behavior, mechanical forces and physical properties acting upon cells [31,91]. Recently, the role of the ECM and tissue biomechanics in cancer progression has also been elucidated. Bi-directional cell-ECM signaling is an integral part of cell behavior in PDAC, triggering oncogenesis and influencing cell fate. Despite this, modeling these biomechanical effects can be difficult, particularly when using stiff 2D substrates such as tissue culture plastic in vitro [92]. New advances in culture systems such as 3D organotypic matrices [93] and organoid culture [94] has helped improve the physiological relevance of research findings relating to mechanobiology, particularly when including stromal components such as CAFs in cancer models. As such, it is prudent to always take into account the model systems used in biomechanical studies as this can influence the findings significantly.

Tyrosine kinase/Ras signaling is one of the main regulators of cell mechanics and an integral element in the reprogramming of normal cells into tumor cells upon KRAS mutation. For example, Panciera et al., (2020) reported that reprograming of normal cells into tumor precursors can also require enhanced ECM stiffness and oncogenic mechanosignaling [95]. Here, increased cytoskeletal tension and cell stiffening triggered activation of YAP/TAZ signaling leading to downstream oncogenic transcriptional responses [95]. Similarly, YAP mechano-response was shown to be positively regulated by the integral membrane protein Caveolin-1 (CAV1) on stiff substrates through an actin dependent mechanism driving acinar-to-ductal metaplasia (ADM) in pancreatitis, a benign inflammatory pancreatic disease [96]. 

**Figure 3:**
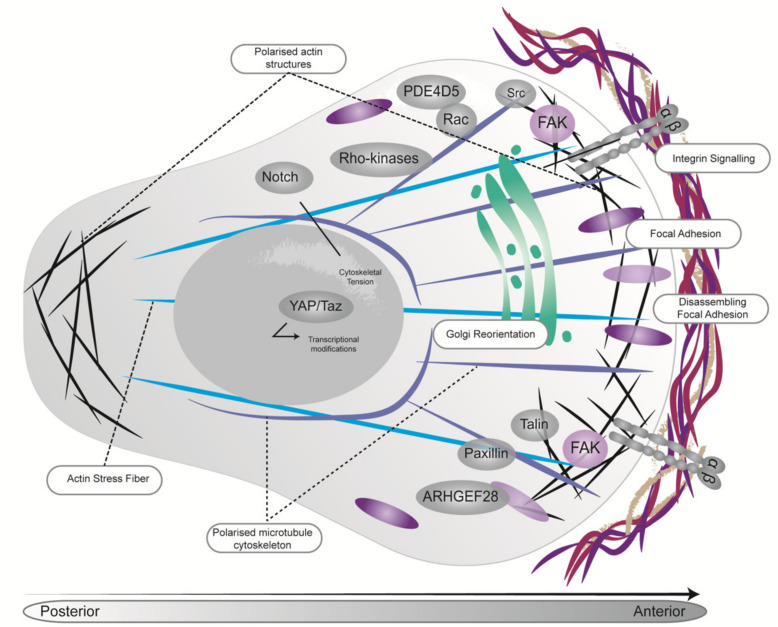
Biomechanical changes in PDAC. Cell-ECM signaling during tumor invasion and migration is sensed and regulated by mechanoreceptors such as integrins. Integrin-stimulated mechanosignaling via the FAK/Src signaling axis leads to downstream activation of Rac, Rho-kinases, Paxillin and Talin. Alteration of these pathways influences Golgi reorientation to the leading edge of migrating cells, polarization of the microtubule and actin cytoskeletons as well as actin stress fibres to establish an anterior-posterior gradient, driving cell migration.

Stiffening of the tumor ECM is regulated and sensed by mechanoreceptors such as integrins, which physically connect cells to the ECM and can stimulate multiple intracellular mechanosignaling proteins such as Rho-associated kinase (ROCK), FAK, RhoA, JAK/STAT and PAK (Figure 3). It has been shown that early targeting (priming) of ROCK activity can impair coordinated cell migration in both in vitro and in vivo models of PDAC [88,97,98]. This is possibly due to the influence of stiffness gradients on tumor cell behavior [88,97,98]. Further analysis of the ECM architecture demonstrated that ROCK inhibition reduced ECM remodeling and subsequently tissue stiffness, influencing downstream signaling and depriving cancer cells of normal or physiological mechano-stimulation [88]. Furthermore, ROCK-mediated collagen remodeling has been implicated in overcoming three-dimensional (3D) stromal constraints, enabling proliferation of PDAC cells [88]. The enzyme lysyl oxidase (LOX) has also been shown to regulate collagen crosslinking and biogenesis and is overexpressed in hypoxic environments, including that found in the KPC mouse model of PDAC [99,100]. In mice bearing early stage primary KPC tumors, combination therapy of a LOX blocking antibody with gemcitabine, decreased matrix crosslinking, thereby reducing metastasis and increasing survival compared to chemotherapy alone [99].

It has been well documented that FAK phosphorylation is a key step in the mechanosensory process [101]. During migration on a flexible substrate, normal fibroblasts were shown to migrate towards a stiffer substrate whilst FAK-null cells showed no preference for soft or stiff substrates. This was thought to be due to the involvement of FAK signaling in cell-substrate adhesion strength, with adhesions at the leading edge responding in a FAK-dependent manner to a more rigid substrate, subsequently pulling cells in the direction of stiffer substrates [102] (Figure 3). This ability to respond to changing matrix forces was abolished in FAK-null fibroblasts where cells failed to show a similar focal adhesion response, highlighting the importance of FAK in responding to physical cues in the TME [102]. A recent study by Jiang and colleagues (2016) demonstrated that FAK signaling is in part responsible for driving the desmoplastic PDAC microenvironment, and that FAK inhibition can reduce the fibrotic and immunosuppressive aspects of the TME. These changes in the TME were shown to sensitize PDAC to immunotherapy and chemotherapy leading to disease stabilization upon combination therapy [103]. However, periods of disease stabilization were followed by the acquisition of treatment resistance and tumor progression [104]. Here, following prolonged treatment with FAK inhibitors, FAK-independent growth was observed, and attributed to a hyperactivation of STAT3 signaling due to loss of stromal TGF-β [104]. Upregulation of such signaling pathways and enhanced protein activation is not exclusive to PDAC. Indeed, similar tumor-stroma crosstalk and mechanical alterations have been reported in breast cancer, melanoma and glioblastoma [105,106,107,108,109]. Overall, these studies highlight the therapeutic potential, but also current limitations of agents designed to disrupt tumor-stroma mechano-reciprocity in PDAC as well as other cancers. 

The integrin signaling axis bridges signaling between the ECM to the contractile actin cytoskeleton, transducing bi-directional responses between cancer cells and stroma in order to guide cellular fate [110] (Figure 3). This is exemplified in a recent study by Chastney et al., (2020), where multiplexed proximity biotinylation was used to assess the adhesome (integrin adhesion complexes) in mouse fibroblasts. This in-depth analysis generated a defined network of adhesome-related associations, which provided unique insight into adhesome components, including new information about spatial and topological organization [111]. Furthermore, a recent study also reported that cancer cells preferentially bind to fibroblast-associated fibronectin via integrin α5β1, in turn triggering enhanced cell migration along the fibronectin fibres [112]. Here, integrin α5β1, stimulated by fibroblasts in the ECM, recruited FAK to focal adhesion sites, which led to downstream activation of B-Raf and Erk [112]. This resulted in subsequent induction of mitogenic signaling and cell proliferation [112]. Additionally, extrinsic and intrinsic integrin-mediated mechanosignaling pathways, via integrin α5, F-actin-YAP-Notch signaling axis, have been shown to coordinate the cell fate of pancreatic progenitors [113]. Furthermore, integrin α5β6 promotes PDAC growth through cell and TME mechanisms and inhibiting this via antibodies enhanced survival by suppressing the pro-tumorigenic TME in PDAC mouse models [114]. Finally, cell attachment to the basement membrane via integrin β1, a mediator of ECM contact, provides a survival advantage over cells lacking this adhesion upon treatment with MEK inhibitors [115]. Overall, these studies highlight that whilst integrin-mediated mechanosignaling drives disease progression, it can also contribute to treatment resistance and presents a promising target for therapeutic intervention.

## 5. Emerging role of Biomechanics Influencing PDAC Metabolism

Cell proliferation and differentiation require the metabolism of nutrients for both energy and biosynthesis of macromolecules. CAFs exhibit diverse functions to sustain tumor growth including providing metabolic support to enable neoplastic proliferation. Recently, a connection between tissue mechanics and cell metabolism has been identified [91,116]. The mechanics and alterations in tumor stiffness were shown to influence the creatine-phosphagen ATP-recycling system, affecting ATP/ADP and ATP/AMP levels, and this was shown to play a role in tumor invasion, migration and metastasis of cancer cells [117]. In addition to regulating cell metabolism via response to mechanosignaling, stromal cells can also directly influence cancer cell metabolism. For example, CAFs support PDAC survival by mediating the effects of extracellular Netrin G1 (a lipid-anchored protein) on glutamate/glutamine metabolism [118]. Interestingly, Netrin G1+ CAFs are intrinsically immunosuppressive, inhibiting natural killer (NK) cell-mediated killing of tumor cells. Inhibition of these metabolic proteins in CAFs has the potential to alter their immunosuppressive capacity, highlighting a link between cell metabolism and tumor immunomodulatory functions. In this context, activation of cytokine receptors by polarised CD4+ T cell-derived cytokines, mediates JAK-STAT signaling directly by upregulating cMyc and driving metabolic reprogramming [119]. This paracrine signaling loop underscores the crosstalk between various cells in the PDAC TME and may provide novel therapeutic targets. Moreover, recent work has revealed metabolomic differences between cancer cells and fibroblasts in PDAC, where isotope labelled nutrients showed that tumor cells exhibit increased levels of pyruvate carboxylase compared to fibroblasts [120]. Thus, highlighting the need to separate the metabolic profiles of specific cell populations within the heterogenous tumor. 

It is becoming increasingly apparent that the collagen-rich ECM of PDAC can restrict nutrients and oxygen to the tumor cells. However, altered metabolism of PDAC cells means that this meshwork can also serve as a proline reservoir for cells, promoting their survival under previously nutrient deficient conditions [121]. Similarly, under stromal-rich, nutrient-deprived conditions, the ECM can also serve as a nutrient source for CAFs. Here, the demand for nutrients drives PDAC cell reprogramming of CAF metabolism, dictating internalization of the ECM as a supply of amino acid precursors for CAF secreted BCKAs (branched-chain-α-ketoacid), upon which PDAC cells rely [122]. Moreover, lysophosphatidic acid (LPA), an abundant signaling lipid in the blood, has previously been shown to serve as both a mitogen and chemoattractant for cancer cells thereby driving metastasis via the circulation. This chemotactic gradient leads to Rho-A generated contractile forces, ECM remodeling and cell invasion [123], which has previously been seen in vivo via intravital imaging [124,125]. Furthermore, following a shift in fibroblast lipid metabolism during PDAC development, stroma-derived lysophosphatidylcholines support PDAC cell membrane synthesis, stimulating growth and migration [126]. Together these studies highlight the influence of metabolites within the desmoplastic TME of PDAC and reveal potential therapeutic avenues to target PDAC aggressiveness.

## 6. The Changing Paradigm of Stromal Co-Targeting in PDAC and Future Perspectives

The majority of PDAC patients present with inoperable, locally advanced, or metastatic disease, making systemic chemotherapeutic regimes standard-of-care in this advanced setting. Currently, gemcitabine, gemcitabine/Abraxane or FOLFIRINOX offer limited improvement in survival. In recent years, our enhanced understanding of the intricate PDAC TME, including its cellular and structural components as well as its interactions with the cancer cells, has alluded to several potential therapeutic opportunities to co-target the TME in PDAC [127].

As CAFs are one of the main contributors to tumor development and progression in PDAC, this dynamic stromal cell population offers a promising therapeutic target, despite their known functional heterogeneity. Stromal desmoplasia in PDAC tumors is purported to be a major biochemical and physical barrier to effective drug delivery in the treatment of PDAC. It is thought that the dense, fibrotic ECM blocks drug penetrance via increases in interstitial fluid pressure (IFP) and hypo-vascularisation. This results in reduced treatment efficacy and resistance to current therapeutics. In recent times, there have been many attempts to co-target CAFs to enhance standard-of-care chemotherapy and emerging treatments such as immunotherapy. Generally speaking, CAFs can be targeted directly or by attempting to reprogram them towards a tumor-restraining phenotype. Similarly, there has also been attempts to block or normalize the reciprocal signaling between the CAFs, tumor cells and other cells of the TME to impede tumorigenesis. Thus far, co-targeting CAFs effectively has been challenging, most likely due to a lack of specific CAF markers, as well as their inherent plasticity in vivo.

The hedgehog signaling pathway is one of the most studied stromal co-targets for the treatment of PDAC. In PDAC, activation of the Hedgehog pathway results in CAF activation via paracrine signaling with adjacent cancer cells, leading to aberrant ECM deposition and promotion of tumorigenesis [34,128]. In 2009, Olive et al. elegantly showed that inhibition of the Hedgehog signaling pathway via IPI-926 improved response to Gemcitabine chemotherapy by reducing desmoplasia and increasing tumor vasculature density, allowing for better chemotherapy penetrance [34]. More recently, Steele et al. (2021) reported that pancreatic myCAFs are more susceptible than iCAFs to Hedgehog-dependent activation. Moreover, treatment with a hedgehog pathway antagonist (LDE225) reduced desmoplasia and primary tumor growth [128]. Despite this promising result, chronic inhibition with LDE225 also reduced the number of cytotoxic T cells [128]. This allowed for the expansion of regulatory T cells, thereby increasing immunosuppression in the TME [128]. Conversely, in PDX models, short-term hedgehog signaling inhibition mediated dose-dependent alterations in vasculature patency, ECM architecture and IFP, increasing the permeability of nanoparticle deposition [129]. These studies suggest that short-term or transient treatment schedules using anti-fibrotic agents could potentially increase the efficiency of subsequent therapeutic agents [88,129], while minimizing the adverse effects of chronic long-term treatment, which have also previously been described in genetic studies of stromal ablation [35,36].

Another study by Elahi-Gedwillo et al., (2019) reported that normalization of the stromal microenvironment using the anti-fibrotic agent halofuginone resulted in disruption of the matrix to improve drug distribution through decreased fibroblast activation [130]. Concomitantly, halofuginone also influenced the immune landscape, allowing greater influx of anti-tumorigenic macrophages and cytotoxic T cells, triggering intratumoral tumor cell death and reducing overall tumor volume [130]. Interestingly, stromal markers such as FAP have been co-opted to produce chimeric antigen receptor T (CAR T) cells for the treatment of PDAC. Multiple studies have shown that FAP-specific CAR T cells can induce an immune response, impeding PDAC tumorigenesis in vivo [131,132,133]. However, in one study bone toxicity and cachexia was observed in the treated animals [131] highlighting the potential dangers of targeting stromal proteins which are not exclusively expressed by CAFs. Moreover, as mentioned previously, Feig et al. (2013) showed that targeting the stroma, in this case via CXCL12, could improve the efficacy of anti-PD1 checkpoint inhibition by improving T cell infiltration [87]. Evidently, more pre-clinical and clinical research is required to fully understand the potential of targeting the stroma to improve the immunogenicity and therefore responsiveness of PDAC tumors to immuno-based treatments.

In the clinic, resection of locally advanced disease is often prevented by the encasement of major mesenteric vessels by the dense ECM. Neoadjuvant chemotherapy in combination with a monoclonal antibody against connective tissue growth factor (pamrevlumab) holds promise for improving resection rates in patients with locally advanced PDAC, as a pre-clinical study showed this combination reduced the dense and fibrotic encapsulation of critical blood vessels [134]. Furthermore, in pre-clinical mouse models of PDAC, the heparan-based mimetic necuparanib exhibits multi-targeting anti-tumor activity, reducing proliferation and invasion in vitro and extending survival as well as reducing metastases in vivo [135]. Interestingly, analysis of plasma samples from patients receiving this treatment, revealed increased levels of ECM remodeling enzymes matrix metalloproteinase 1 (MMP1) and tissue inhibitor of metalloproteinase 3 (TIMP3), eluding to an ECM remodeling mechanism [135]. Moreover, other agents targeting MMPs in the context of gemcitabine/Abraxane treatment in advanced PDAC patients have demonstrated favorable safety profiles and clinical activity and could prove promising in future treatment of metastatic disease [136]. However, despite promising pre-clinical results, clinical targeting of the fibrotic ECM (specifically hyaluronidase with PEGPH20) in the context of gemcitabine/Abraxane or FOLFIRINOX chemotherapy has yielded conflicting results [137,138]. Given the complexity of the PDAC TME, further understanding is required to improve current stromal therapies and to fine-tune the balance and timing of stromal co-targeting.

In recent times, nanomedicines have begun to emerge as a new treatment modality for cancer patients, owing to their high tissue specificity, excellent pharmacokinetics, therapeutic efficiency, and minimal side effects. Recently, nanomedicines have been manufactured to exquisitely modulate unique aspects of the TME, demonstrating highly effective anti-tumor and anti-metastatic properties, whilst also enhancing drug efficiency [139,140]. For example, targeting the collagen-specific molecular chaperon, heat shock protein 47, on stromal cells via nanoparticle delivery or siRNA can regulate the TME by inducing quiescence, inhibiting fibrosis and enhancing subsequent chemotherapy [141]. Similarly, Sharbeen et al., (2021) utilized a siRNA nanoparticle and clinical-grade pharmacological inhibitor (sulfasalazine) to target the amino acid transporter SLC7A11 in PDAC [142]. In this study, inhibition of SLC7A11 via the siRNA nanoparticle in patient-derived PDAC specimens resulted in marked anti-tumorigenic effects, including normalization of stromal desmoplasia [142]. Furthermore, nanomedicines against ECM biomarkers could have a dual function in both clinical imaging and drug transportation to disease sites. For example, immuno-PET/CT imaging of a nanobody (NJB2) for a disease-specific alternatively spliced domain of fibronectin was used to detect primary and metastatic cancer sites with high specificity, including detection of early pancreatic lesions [143]. The high specificity of NJB2 renders it a promising candidate for nanoparticle-based therapy. Overall, the restoration of stromal homeostasis by nanoparticles represents another exciting novel approach to improve the efficacy of chemotherapy and other agents in stroma-rich tumors.

## 7. Concluding Remarks

**Figure 4:**
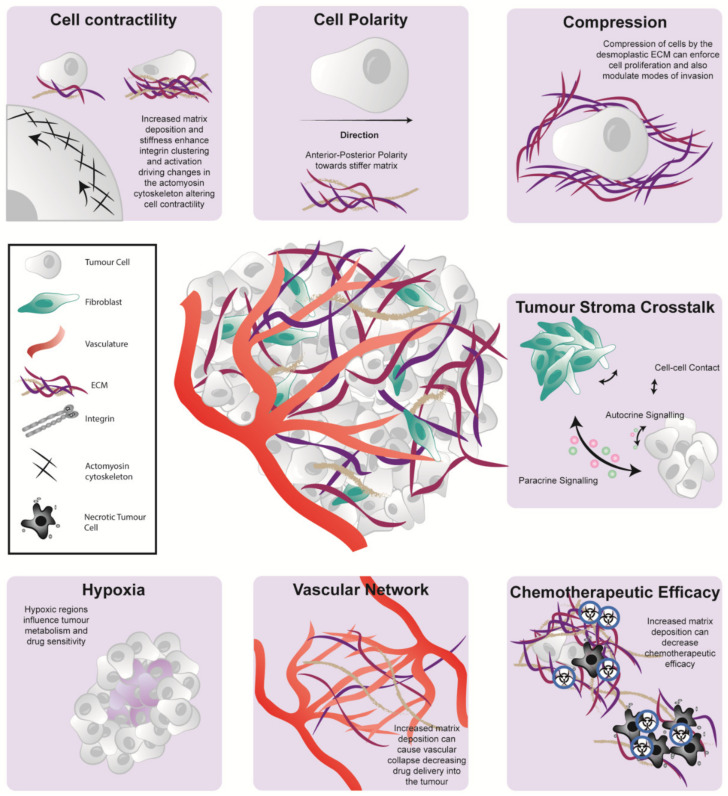
The pancreatic microenvironment influences tumorigenesis via a range of biochemical and biomechanical phenomena. The pancreatic TME has multiple influences on cell be havior including cell contractility (top left), cell polarity (top middle), the generation of compression forces (top right) and tumor-stroma crosstalk via direct cell-cell contact, paracrine or autocrine signaling (middle right). It can also cause the generation of hypoxic tissue pockets (bottom left), modulation of the tumor vascular network (bottom middle) and diminished cancer cell vulnerability to chemotherapy (bottom right).

The cellular and architectural compartments of the PDAC TME play a significant role in disease development, progression, and therapeutic response (Figure 4). Here, we discussed recent studies which highlighted the unique and individual nature of the PDAC stroma and its role in contributing to tumor heterogeneity and patient prognosis. Despite the identification of several promising targets to modulate the TME, they are yet to show meaningful improvement in the clinical outcome of disease beyond early phase clinical trials. The conflicting results of several stromal targeting studies show the double-edge sword (favourable and unfavourable aspects) of stromal co-targeting; where elimination of the stromal barriers that influence the delivery of chemotherapeutic agents can also potentially drive tumor progression. As such, there is an imperative need to understand the complex role of the PDAC TME to improve stromal co-targeting regimes and enhance patient survival.
